# Wound Debridement with Copper Oxide Dressings: Bridging the Gap Between Clinical Observations and the Basic Science Underlying Endogenous Autolysis

**DOI:** 10.3390/jfb17070350

**Published:** 2026-07-18

**Authors:** Eyal Melamed, Ithamar Cheyne

**Affiliations:** 1 Rambam Health Care Campus, Haifa 3109601, Israel; 2 Bnai-Zion Medical Center, Haifa 33394, Israel; 3 School of Medicine, Medical University of Warsaw, 02-091 Warsaw, Poland; ithamar1512@gmail.com

**Keywords:** debridement, copper oxide dressings, biomaterials, copper oxide microparticles, angiogenesis, matrix metalloproteinases, endogenous autolysis, wound healing, tissue remodeling

## Abstract

Objective: The usefulness of available wound debridement strategies, including endogenous autolysis and enzymatic, larval, and surgical approaches, is often limited by poor wound bed biology, availability, and invasiveness. Although copper oxide-containing dressings (CODs) have demonstrated broad wound-healing activity in basic science and clinical studies, their potential to modulate debridement has not been specifically characterized. Methods: We conducted a retrospective analysis of five severe clinical cases (six limbs) characterized with extensive necrosis, impaired perfusion, and frequently compromised systemic conditions. Sequential clinical imaging and detailed follow-up were used to assess wound bed dynamics. All cases demonstrated a period of clinical stagnation prior to COD initiation under standard wound care, ranging from 1–4 weeks in the acute cases to approximately 8 years in the chronic venous ulcer, allowing within-case temporal comparison of wound behavior before and after COD introduction, during which no other major changes in local wound management were made. Observations were interpreted in the context of relevant published basic science. Results: In five of the six limbs, major amputation or revision to a higher level had been indicated prior to COD initiation. In all cases, application of COD was associated with rapid and extensive clearance of devitalized tissue, with major wound-bed changes observed within 2–6 weeks, consistent with activation of endogenous autolytic mechanisms. Relevant published literature identifies copper-dependent pathways (CDPs), including the MMP–TIMP axis, inflammatory signaling via NF-κB, and macrophage polarization, that may provide a biologically plausible explanation for these clinical observations. Analysis of serial photographs demonstrated concurrent emergence of granulation tissue and vascularization, supporting synergism among angiogenesis, granulation tissue formation, and debridement, all induced by COD. Conclusions: The observations support the hypothesis that copper ions and CODs may amplify autolytic tissue clearance through CDPs, with concurrent synergistic interaction between angiogenesis and debridement.

## 1. Introduction

Good tissue quality is essential for wound healing, yet many wounds are characterized by compromised viability and necrosis, which impede repair [1]. Debridement is therefore a cornerstone of wound management, conducted with the aim of removing non-viable tissue and restoring a healing-permissive environment [2]. Although surgical debridement is effective, it is not always feasible and may damage viable tissue, leaving many wounds reliant on endogenous autolytic and enzymatic mechanisms [2].

Copper cations appear to engage multiple interconnected molecular and cellular pathways involved in necrotic tissue autolysis. By modulating extracellular matrix remodeling via the MMP–TIMP axis, activating controlled inflammatory signaling via NF-κB, and polarizing macrophages, copper cations enhance the debridement response [3,4,5,6,7,8].

These physiological debridement processes are often insufficient in the presence of extensive necrosis, impaired perfusion, hypoxia, or systemic illness, leading to insufficient inflammation, bacterial persistence, and delayed healing [9,10]. This creates a clear clinical need for therapies that directly enhance, rather than merely support, native debridement pathways.

Copper-based wound dressings are engineered as composite biomaterials in which copper oxide (CuO or Cu_2_O) nanoparticles (NPs) or microparticles (MPs), or metal–organic framework nanoparticles (Cu-MOF NPs), are integrated into polymer matrices to enable controlled, dose-regulated release of cupric (Cu^2+^) or cuprous (Cu^+^) ions, respectively. Copper loading varies considerably across formulations, ranging from 0.2–1.6 wt% CuO in electrospun core–shell nanofibers comprising a polycaprolactone core and sulfosuccinic acid–polyvinyl alcohol shell [11] to 20% tannic acid/Cu^2+^ crosslinks in carboxymethyl chitosan hydrogels [12] and up to 10 wt% CuO in tragacanth/chitosan systems [13], with polymersome-loaded Pluronic F127 gels maintained at ~50 µg/mL CuO-equivalent to balance efficacy and safety [14]. The polymer scaffold governs release kinetics: hydrophilic matrices such as thermoplastic starch/polyvinyl alcohol/cellulose nanocrystal films [15] and swelling hydrogels [13] drive diffusion-mediated release over hours to days.

While numerous experimental copper-containing biomaterials have been described in the literature, current clinical experience indicates that cuprous oxide-impregnated dressings (CODs) by MedCu^®^ (MedCu Technologies ltd, Herzliya, Israel) are, to the best of available knowledge, the only copper-containing wound dressings in routine human clinical use [16]. These copper oxide dressings are engineered as non-woven, multilayer constructs in which the polymer matrix itself serves as the structural reservoir, with cuprous oxide microparticles incorporated at approximately 2.65% (*w*/*w*) into both the external spunbond nonwoven layer that contacts the wound bed and the inner absorbent core [16,17]. The use of non-soluble cuprous oxide microparticles embedded within the fiber network confers mechanical stability whilst enabling sustained ion leaching at parts-per-million levels over at least seven days, avoiding bulk particle dissolution whilst delivering bioactive Cu^2+^ continuously to the wound microenvironment [11,17,18]. The absorbent inner layer simultaneously manages exudate and provides the moisture necessary to drive copper ion release from the solid-phase reservoir, creating a self-regulating, moisture-dependent delivery system [17].

CODs represent a novel approach [16]. Experimental and clinical studies show that copper cations activate key wound-healing pathways, including antimicrobial activity (including biofilm destruction), modulation of inflammation, angiogenesis, and enzymatic tissue remodeling [3,16,19,20]. Copper stabilizes hypoxia-inducible factor (HIF-1α), leading to upregulation of pro-angiogenic and reparative mediators such as VEGF and PDGF, thereby promoting granulation tissue formation and epithelialization [3,21].

To date, debridement has not been formally recognized as a functional property of copper-containing dressings. However, clinical observations of rapid clearance of devitalized tissue following COD use prompted us to explore potential mechanistic explanations for this effect [22,23]. In this paper, we analyze five highly compromised wounds to demonstrate preclinical concepts in clinical practice. We examine sequential images, paying special attention to the inter-relationship between the disappearance of necrotic tissue and angiogenesis and granulation tissue formation.

## 2. Materials and Methods

Beginning in May 2019, CODs were introduced into clinical practice at Rambam Health Care Campus. Initial use was limited to stable patients with hard-to-heal wounds, primarily diabetic foot ulcers. With accumulating clinical experience and emerging supportive evidence, indications were progressively expanded to include complex and severe wounds, including cases with extensive necrosis, compromised perfusion, and failure of conventional surgical and non-surgical treatments.

Cases were purposively selected based on two baseline features present before COD initiation—extensively devitalized or necrotic tissue and the absence of clinical evidence of overt invasive infection—together with adequate serial photographic documentation to follow wound-bed evolution. In most cases, copper dressings were introduced only after conventional management had failed. The bilateral open-fracture case was managed throughout by a separate surgical team. The purpose of this series was to characterize the clinical debridement pattern and its temporal relationship with granulation-tissue formation, not to estimate an overall response rate to COD.

This report analyzes a retrospective case series of six limbs in five patients, with, with diverse wound etiologies, receiving inpatient (*n* = 4) or outpatient (*n* = 1) treatment, between 2021 and 2024. All wounds demonstrated clinical stagnation with devitalized or necrotic tissue not responding to standard wound care treatment (Table 1) with five of six limbs considered for major amputation or higher-level revision. This clinical course allowed for within-case temporal comparison of wound behavior before and after the initiation of COD treatment, during which no other major changes in local wound management were introduced.

Care was taken to ensure coverage of all wound surfaces and cavities, as well as normal skin surrounding the wound. The dressings were changed by healthcare personnel once or twice weekly throughout all stages of wound healing. No adjunctive enzymatic or chemical debridement agents were used and no new antibiotic was initiated.

Clinical analysis using high-quality sequential images taken at every dressing change focused on the extent and rate of necrotic tissue clearance, granulation tissue formation, local inflammatory signs, wound demarcation, and readiness for definitive closure or grafting.

As this was a retrospective case series describing COD treatment provided as part of routine departmental care rather than under a research protocol, the requirement for formal IRB approval was waived by the Institutional Review Board of Rambam Health Care Campus.

## 3. Results

The key clinical characteristics and outcomes of the five cases are summarized in Table 1 and Table 2.

### 3.1. Case 1—Chronic Venous Leg Ulcer in a Patient with APLA Syndrome

A 48-year-old male with antiphospholipid antibody (APLA) syndrome and chronic venous insufficiency following recurrent venous thrombosis presented with a chronic venous leg ulcer of approximately eight years’ duration. Over this period, the patient had been treated by experienced multidisciplinary wound care teams without meaningful clinical improvement, and amputation was offered as an option.

**Figure 1:**
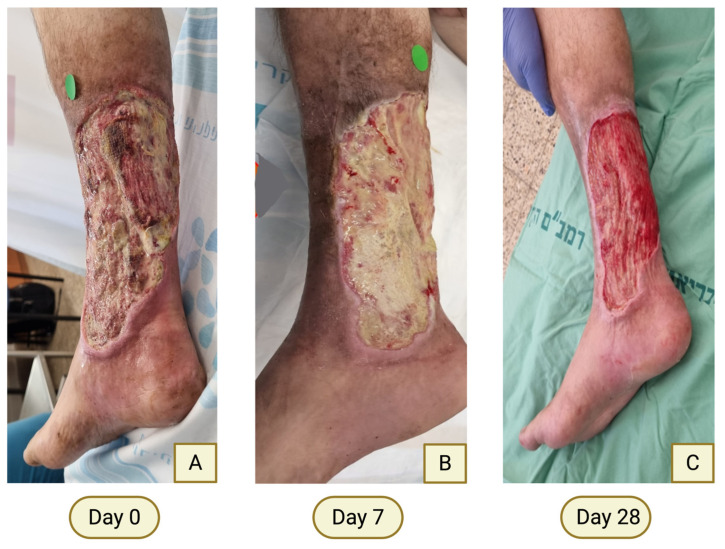
Sequential images demonstrate progressive debridement of a chronic (eight-year) venous leg ulcer in a patient with antiphospholipid antibody (APLA) syndrome. (**A**) Baseline appearance of the wound, measuring approximately 185 × 120 mm, predominantly covered by fibrinous and devitalized tissue. (**B**) One week after initiation of copper oxide dressings (CODs), partial clearance of non-viable tissue with early foci of granulation tissue is observed. (**C**) Four weeks after COD initiation, the wound bed is largely covered by granulation tissue, with thin residual fibrinous tissue over the medial malleolus.

On examination, the ulcer was extensive, measuring 185 × 120 mm, and characterized by near-complete coverage with non-viable tissue and minimal-to-absent granulation tissue. Peripheral arterial pulses were palpable and symmetrical, with no clinical signs of arterial insufficiency. Treatment with COD (changed weekly) was initiated. Progressive autolytic debridement was observed at each weekly dressing change, accompanied by early granulation tissue formation. After four weeks of COD treatment, the wound bed demonstrated complete clearance of devitalized tissue and 100% granulation tissue coverage. The progression of the wound is depicted in Figure 1.

### 3.2. Case 2—Debridement of Marginal Wound (Stump) Necrosis

A 60-year-old woman with diabetes mellitus was admitted following a supracondylar femoral fracture. She initially underwent open reduction and internal fixation with a plate. The surgical site subsequently became infected, necessitating removal of the plate and application of external fixation. Despite prolonged management, fracture healing failed and wound complications persisted. Ultimately, a transfemoral amputation above the fracture level was performed.

Postoperatively, the amputation stump failed to heal and required revision surgery, which also failed to achieve wound healing. Hip disarticulation was therefore considered.

**Figure 2:**
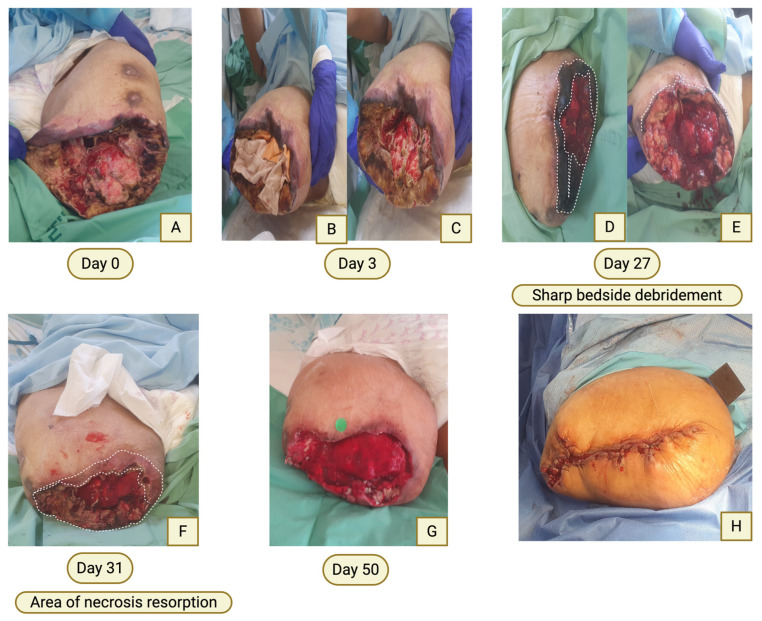
Progressive marginal and central debridement with granulation following COD therapy. (**A**) At presentation, the amputation stump shows partial central granulation surrounded by necrotic and ischemic wound margins. (**B**,**C**) Early changes within several days of COD initiation, including increased central granulation and clearer demarcation between viable and non-viable tissue. (**D**) Day 27: White dashed lines outline necrotic wound margins prior to bedside intervention. (**E**) Immediate appearance following limited bedside sharp marginal debridement (~2 mm) to prevent pain and preserve viable skin. (**F**) Resolution of previously necrotic areas (dashed lines). (**G**) Day 50: Fully viable wound margins with complete granulation tissue coverage, allowing delayed primary closure (**H**).

At presentation, the stump demonstrated partial (pink) central granulation tissue surrounded by necrotic and ischemic wound margins. Within several days of COD initiation, the central granulation tissue became more extensive and robust, while a clearer demarcation developed between viable and non-viable tissue at the wound margins. After 4 weeks (day 27), bedside sharp marginal debridement was performed without anesthesia, limited to approximately 2 mm from the necrotic wound edges, thereby eliminating pain and minimizing damage to viable skin. Four days later, most residual necrotic tissue had been resolved. Within two to three weeks, the wound demonstrated fully viable margins and complete granulation tissue coverage, allowing delayed primary closure at day 52. The progression of the wound is depicted in Figure 2.

### 3.3. Case 3—Biological Reactivation of a Critically Ischemic Amputation Stump

One month after coronary artery bypass grafting, during which a saphenous vein graft was harvested from the lower limb, a 71-year-old male presented with complications at the graft harvest site. This site became severely infected and ischemic, ultimately necessitating below-knee amputation followed by progression to above-knee amputation. Due to ischemia, the stump was closed loosely but failed to heal. The patient was critically ill, mechanically ventilated, and systemically compromised, with additional pressure-related wounds. The thigh was not warm or edematous (failure to raise an inflammatory response). The sutures were removed, revealing an entirely non-viable stump with no inflammatory response, including erythema and edema. CODs were then applied and replaced twice weekly. As early as the first dressing change, early granulation tissue was observed, with progressive autolytic debridement alongside increasing granulation tissue during subsequent changes. At the fourth change, 14 days after initiation of COD therapy, a significant purulent-appearing discharge developed. No new thigh swelling or erythema was observed and preserved skin wrinkling was visible. In view of the continuing development of abundant granulation tissue and the absence of concurrent clinical deterioration, COD treatment was continued with close clinical observation. Revision amputation remained a possible alternative; however, there was no urgent clinical indication for repeat surgery at that stage, and the patient’s severe general condition made further surgery particularly high risk.

Three days later, no purulent discharge was observed and the wound bed appeared dry, with organized, cauliflower-like granulation tissue and clean margins. It is noteworthy that such granulation tissue appears paler. This may reflects maturation of the granulation tissue, with increasing extracellular matrix deposition, including fibroblasts and collagen, and a relatively less prominent vascular appearance compared with earlier, highly vascular granulation tissue [24]. The green discoloration of the wound surface reflects *Pseudomonas aeruginosa* pigments (pyocyanin/pyoverdine) in the exudate, consistent with culture-proven colonization [25,26].

**Figure 3:**
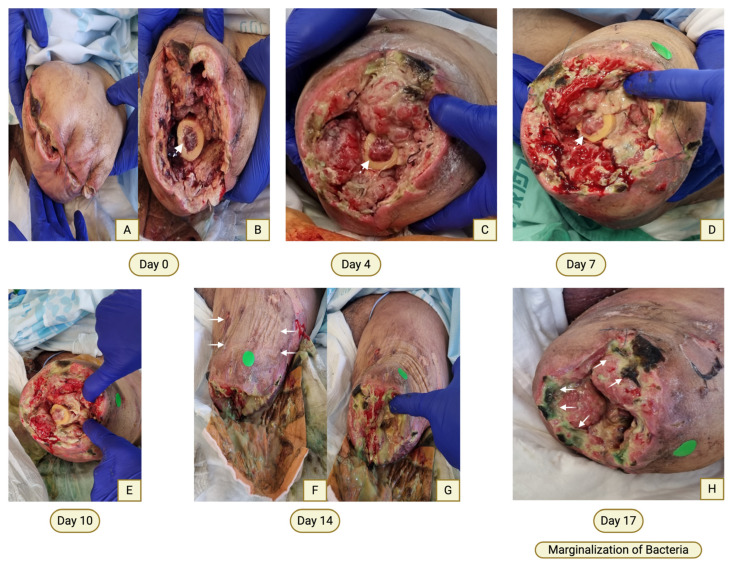
Sequential images demonstrate a transition from ischemic, biologically inactive tissue to robust granulation with a controlled inflammatory response. (**A**,**B**) A transfemoral amputation stump in a critically ill patient following coronary artery bypass surgery. Images taken before and after suture removal, showing an entirely non-viable stump with extensive devitalized tissue, exposed bone, and the absence of surrounding erythema or edema. (**C**–**E**) Twice-weekly COD changes demonstrating progressive replacement of necrotic tissue by granulation tissue, including involvement of exposed bone marrow (arrowheads). (**F**,**G**) Day 14 (fourth dressing change): purulent-appearing discharge within the wound bed without accompanying clinical signs of infection (no thigh swelling or erythema; white arrows indicate preserved skin wrinkling). (**H**) Resolution of discharge with a dry wound bed, solid and organized cauliflower-like granulation tissue, and clean, viable margins three days later. The cauliflower-like tissue is denser and appears paler, likely due to deposition of extracellular matrix and fibroblasts. Residual surface microbial debris, presumably Pseudomonas pigment (pyocyanin/pyoverdine), is, localized toward the wound margins (white arrows).

The patient succumbed shortly afterward due to his cardiopulmonary complications. The progression of the wound is depicted in Figure 3.

### 3.4. Case 4—Debridement of Extensive Eschar Following Electrical Burn

**Figure 4:**
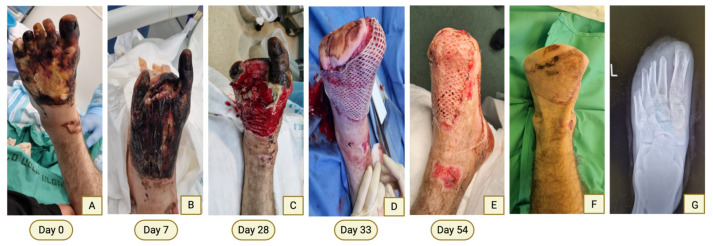
Clinical course following electrical injury to the left foot. (**A**) Extensive necrosis involving the dorsal foot and toes two weeks post injury. COD treatment was initiated. (**B**) Evolution to eschar formation with subsequent amputation of middle toes. (**C**) After three additional weeks of COD therapy: progressive autolytic debridement with replacement by dense, well-vascularized granulation tissue. (**D**) Definitive surgical management: trans-metatarsal amputation with preservation of plantar skin and split-thickness skin grafting onto a well-prepared wound bed. (**E**,**F**) Complete graft taken two weeks and two months post grafting. (**G**) Corresponding radiographic image.

A 19-year-old patient sustained an electrical injury, resulting in a burn to the left foot. The patient was initially treated with betadine-soaked dressing, followed by chlorine-based solutions and sulfurous (mafenide acetate) application. The devitalized tissue, mainly on the dorsum of the foot and toes, underwent denaturation to necrotic eschar or dry gangrene of the toes. The use of COD began on the 10th day after injury. The left foot demonstrated extensive necrosis of the toes, including the first metatarsal head and the dorsum of the foot. Surgical resection of the necrotic middle toes was performed two weeks after injury, COD was applied directly to the open wound bed and fresh surgical margins, with coverage of the surrounding skin, leaving the definitive amputation of the hallux and the fifth toe (and skin grafting) to a later stage. COD was changed once weekly. Progressive autolytic debridement was observed, with gradual and steady replacement of necrotic tissue by thick, red granulation tissue. The surgical intervention consisted of a transmetatarsal amputation to preserve good-quality plantar skin at the forefoot and split-thickness skin grafting onto the dense, well-vascularized granulation tissue. The graft demonstrated complete take, consistent with prior successful wound bed preparation. The progression of the wound is depicted in Figure 4.

### 3.5. Case 5—Debridement and Revascularization of Extensive Devitalized Tissue Following an Open Fracture

A 73-year-old man with a 25-year history of diabetes mellitus was injured in a farming accident, causing a high-energy open fracture of the left femur and an open dislocation of the right knee. The injury was accompanied by extensive contamination of the deep soft tissues with earth and animal fecal material.

Urgent CT angiography demonstrated patent major arteries in both limbs. Emergency surgery included irrigation, sharp debridement, and the application of external fixators. In the ICU and then in the orthopedic ward, the wounds were managed with chlorine-based solutions, which were temporarily replaced with mafenide acetate and then resumed. Routine antibiotics (cefazolin and gentamicin) were used initially, followed by minocycline, levofloxacin, and piperacillin/tazobactam.

**Figure 5:**
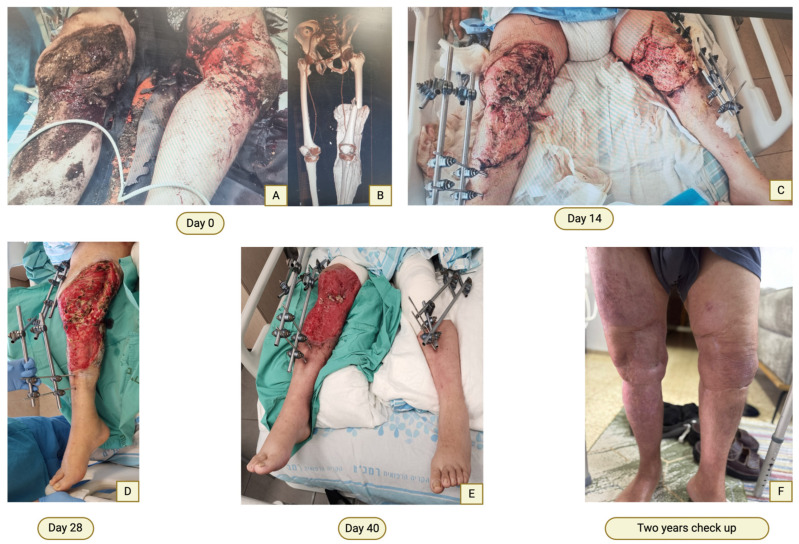
Progressive debridement and wound bed reactivation following COD therapy in a contaminated open fracture. (**A**) Day 0: extensive soft tissue devitalization following high-energy open fracture with severe contamination. (**B**) CT angiography demonstrates skeletal injury with preserved arterial perfusion. (**C**) Day 14: persistent devitalized tissue despite prior surgical debridement and antiseptic wound care. (**D**) Day 28: marked replacement of necrotic tissue by dense granulation tissue following COD therapy. (**E**) Day 40: well-vascularized wound bed suitable for skin grafting. (**F**) Long-term follow-up demonstrating stable wound closure and limb preservation.

Two weeks post injury, the wounds remained extensively devitalized without clinical progression. Given the extent of necrosis and long-standing diabetes, bilateral trans-femoral amputation was planned. Instead, wound management was replaced with COD, applied twice weekly directly to the wounds and surrounding skin. At the first dressing change, significant purulent discharge was observed bilaterally. Although this finding raised concern for infection, there was no concurrent clinical deterioration or other local signs suggesting progressive infection, and progressive granulation tissue formation was evident. As in Case 3, the overall clinical course suggested a possible inflammatory or debridement-related phase rather than progressive infection, and COD treatment was therefore continued under close clinical observation. Subsequent dressing changes showed progressive autolytic debridement, with necrotic tissue replaced by abundant, well-vascularized granulation tissue and complete resolution of purulent discharge. Within three weeks of COD therapy, approximately 90% of the wound bed was covered with granulation tissue. Split-thickness skin grafting was performed 8–10 weeks post-injury. As of a two-year follow-up, the patient walks with a walker due to arthritic changes in the knee, but the skin is intact. The progression of the wound is depicted in Figure 5.

## 4. Discussion

This case series is presented as a hypothesis-generating translational analysis addressing a gap between established biological knowledge and clinical application. Although copper-dependent mechanisms involved in wound healing and tissue remodeling are well described, their deliberate clinical utilization to enhance endogenous autolytic debridement has not been previously explored. Across five severe, end-stage wounds (with four of five considered for amputation), initiation of copper oxide-containing dressings was followed by a marked clinical shift toward autolytic debridement and granulation tissue development. We therefore reviewed relevant copper-dependent mechanisms described in the literature to propose biologically plausible explanations for these observations.

In general practice, most wounds, except for clean surgical wounds, involve tissue injury, manifesting as compromised, devitalized, or necrotic tissue. Good tissue quality is a prerequisite for effective wound healing. Within the well-established TIME framework for wound healing, tissue management is the first and most critical therapeutic priority [27]. However, debridement, the removal of devitalized tissue to restore a healing-conducive wound environment, is the first and likely most fundamental component of wound bed preparation [2]. This has led some wound specialists to use the acronym DIME instead of TIME, replacing “Tissue” with Debridement” [28,29]. Surgical debridement entails rapid and effective removal of necrotic tissue but is inherently limited by patient tolerance, clinical context, and the risk of collateral damage to viable tissue [2,10]. Physiological selective debridement, although more patient-friendly, is slower and dormant in stagnated wounds. Available solutions for wound bed preparation include agents that condition the wound for physiological debridement (e.g., hydrocolloids, hydrogels), enzymatic dressings (e.g., collagenase), and biological debridement (maggot therapy) [2,9]. Each of these approaches has its pros and cons. In stagnant wounds, one cannot rely on physiological wound debridement; direct stimulation of debridement mechanisms is mandatory. Copper has been shown to modulate autolytic and enzymatic mechanisms that rely on endogenous proteolytic enzymes, macrophage activity, and regulated inflammatory signaling [10], consistent with the clinical observations presented herein, right after COD therapy is initiated.

**Figure 6:**
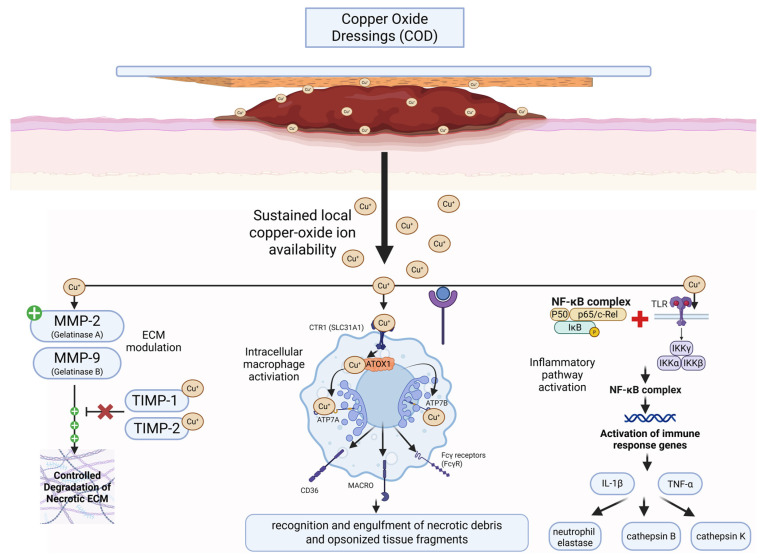
Schematic representation of proposed mechanisms by which copper oxide dressings (CODs) promote active physiological wound debridement. CODs provide sustained local release of copper ions (Cu^+^), which modulate extracellular matrix proteolysis through regulation of MMP–TIMP balance, enhance macrophage activation and phagocytic clearance of necrotic tissue via copper-dependent transport pathways, and regulate NF-κB-mediated inflammatory signaling. Collectively, these coordinated processes facilitate controlled clearance of devitalized tissue while preserving a wound environment conducive to subsequent healing.

Figure 6 depicts the molecular and cellular mechanisms through which CODs promote active physiological wound debridement.

At the extracellular level, copper ions modulate matrix metalloproteinase activity, particularly that of MMP-2 (gelatinase A) and MMP-9 (gelatinase B), enzymes central to the degradation of denatured collagen, fibrin, and necrotic extracellular matrix components. Copper exposure shifts the balance between MMPs and their endogenous inhibitors, tissue inhibitors of metalloproteinases (TIMP-1 and TIMP-2), favoring regulated proteolysis required for effective clearance of devitalized tissue while preventing excessive matrix destruction [3,30,31].

Concurrently, copper ions are taken up by macrophages predominantly via the high-affinity copper transporter CTR1 (SLC31A1). Intracellular copper trafficking is mediated by the chaperone ATOX1 and the ATP-dependent copper exporters ATP7A and ATP7B, enabling copper-dependent macrophage activation and functional polarization. This process enhances the expression of key phagocytic receptors, including CD36, MARCO, and Fcγ receptors (FcγRs), thereby facilitating efficient recognition and engulfment of necrotic debris and opsonized tissue fragments. At the signaling level, copper modulates inflammatory pathways essential for effective debridement [4,32,33,34].

Copper-dependent regulation of NF-κB signaling leads to controlled induction of downstream cytokines such as TNF-α and IL-1β, supporting macrophage recruitment and protease activation without excessive inflammatory amplification. This regulated inflammatory milieu promotes the activation of additional proteolytic enzymes involved in early wound cleaning, including neutrophil elastase and lysosomal cathepsins, particularly cathepsin B and cathepsin K [3,35,36,37,38,39].

Collectively, these convergent pathways result in efficient physiological wound debridement, characterized by controlled clearance of necrotic tissue and preservation of a wound environment necessary for subsequent healing.

This constellation of enzymatic and receptor-mediated effects is consistent with active initiation of physiological debridement, rather than secondary autolysis resulting from passive wound bed conditioning. Whereas conventional dressings primarily optimize environmental parameters, such as moisture balance and exudate control, to permit endogenous debridement when host biology is intact, we propose that COD actively modulates the wound microenvironment to promote proteolytic activity and macrophage-mediated clearance. This mechanistic distinction provides a biologically grounded explanation for COD’s ability to induce effective debridement even in ischemic or biologically inactive wounds, as demonstrated in the cases presented herein.

**Figure 7:**
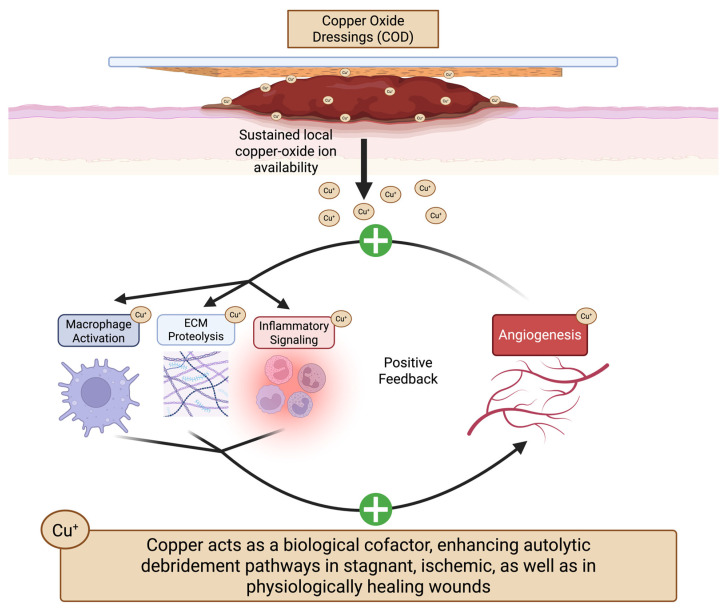
Copper cations appear to engage multiple interconnected molecular and cellular pathways involved in necrotic tissue autolysis. By modulating extracellular matrix remodeling via the MMP–TIMP axis, activating controlled inflammatory signaling via NF-κB, and polarizing macrophages, copper cations enhance the debridement response. Beyond their direct role in physiological debridement, COD and copper cations promote angiogenesis, creating a synergistic interaction in which improved perfusion and tissue regeneration further amplify autolytic tissue clearance. In contrast, conventional debriding dressings (e.g., hydrocolloids) only permit passive autolysis, which is usually insufficient in hard-to-heal wounds, and act slowly in physiological wounds.

As shown in Figure 7, the present case series suggests that CODs induce a coupled positive feedback loop in which the components of wound debridement (macrophage activation, ECM remodeling, and inflammatory signaling control) and tissue regeneration (angiogenesis) amplify each other. Clinically, this is most clearly reflected by the consistent observation that extensive devitalized tissue is eliminated concurrently with the emergence of dense, well-vascularized granulation tissue, as seen in Cases 2–5 and most strikingly in the ischemic stump in Case 3. This synergy is reflected in the rapid emergence of dense granulation tissue and extracellular matrix formation, which in turn provides the biological substrate for the continued production and secretion of autolytic enzymes. This positive feedback loop is reinforced by several copper-dependent biological programs, including matrix remodeling (e.g., MMP–TIMP regulation), controlled inflammatory activation (e.g., NF-κB-mediated signaling), macrophage-mediated clearance, and hypoxia-responsive angiogenesis (e.g., HIF-1α–VEGF signaling) [3,7,8]. The emergence of a systems-level behavior in which CODs synchronously reactivate both sides of wound biology—tissue clearance and tissue reconstruction—is the likely reason for the ability of CODs to overcome the deadlock typical of hard-to-heal wounds.

The clinical evolution of Case 3 supports the idea that CODs actively reinitiate physiological debridement in wounds otherwise locked in biological arrest. The baseline condition (Figure 3A,B) reflects a state of complete tissue non-viability, profound ischemia, and absence of inflammatory signaling, a milieu in which endogenous autolysis and immune-mediated clearance are inactive. Following initiation of COD therapy, the early treatment phase (Figure 3C–E) demonstrates reactivation of coordinated wound-healing pathways, with progressive granulation tissue formation occurring alongside enzymatic degradation and removal of necrotic tissue, consistent with concurrent angiogenesis, matrix remodeling, and immune engagement. Nevertheless, at the following dressing change (Figure 3F), we observed increased volumes of thick exudate resembling pus, with no local signs of infection, serving as a positive sign of robust immune response in the wound bed. In the subsequent phase (Figure 3H), the wound stabilizes into a uniformly granulating bed with peripheral displacement of residual debris, indicating resolution of active debridement and restoration of organized tissue regeneration. Collectively, the sequential biological states depicted in Figure 3A–H illustrate a copper-driven synergy capable of reactivating wound biology despite severe ischemic constraints.

Importantly, purulent-appearing exudate should initially be regarded as potentially infectious and evaluated accordingly. The diagnosis of wound infection relies on the overall clinical assessment and constellation of local and systemic findings rather than on a single wound feature alone [40]. In selected circumstances, as observed in Cases 3 and 5, purulent-appearing exudate may instead reflect an inflammatory or debridement-related phase rather than progressive infection. Such an interpretation should be made cautiously and only after consideration of the overall clinical course and the absence of other features suggestive of invasive infection, including progressive erythema, swelling, advancing necrosis, systemic deterioration, or other concerning local or systemic signs. Case 4 demonstrates a similar pattern, with progressive replacement of eschar and necrotic tissue by granulation tissue over time. A comparable synergistic interaction between debridement and granulation tissue formation is further illustrated in Case 5, entailing a patient with long-standing diabetes mellitus and mangled extremities. No wound progression was observed with abundant devitalized tissue; after two weeks, despite conventional surgical and medical treatment, bilateral lower-limb amputation was deemed necessary. COD therapy was initiated, and healing progressed rapidly, with >90% granulation tissue in both legs after two weeks. Notably, at the first dressing change, thick exudate resembling pus was noted, representing emergence from stagnation.

Large and complex wounds are increasingly treated with negative-pressure wound therapy (NPWT), which primarily promotes angiogenesis and exudate management but lacks inherent debridement activity. In such wounds, COD may offer enhanced biological activity by directly augmenting debridement. In a randomized controlled trial, COD was non-inferior to NPWT for the prespecified primary endpoint of wound-size reduction, using a 20% non-inferiority margin (*p* < 0.01) [22]. However, the trial included diabetic foot wounds after surgical debridement or partial foot amputation and did not evaluate clearance of devitalized tissue as a primary endpoint [22].

### Limitations

The conclusions presented in this manuscript are derived from a retrospective analysis of a small number of extreme clinical cases and are therefore descriptive in nature; thus, causal inference cannot be established. Although the prolonged period of wound stagnation prior to initiation of COD provided an internal comparison, it does not represent a substitute for formal controls. Proposed mechanistic interpretations are grounded in the established biological literature and inferred from observed clinical behavior rather than direct molecular or histological measurements and should thus be regarded as hypothesis-generating. Prospective controlled studies and targeted mechanistic investigations are warranted to test the hypotheses generated via this case series using predefined clinical endpoints and direct biomarker-based assessment.

Additional unmeasured systemic and wound-specific variables, including nutritional status, glycemic control, mechanical loading or offloading, and other aspects of supportive care, may have influenced wound evolution and further limit attribution of the observed changes solely to COD treatment.

Furthermore, the retrospective design precluded standardized quantitative assessment of wound parameters. Although high-quality sequential images were available, reliable planimetric measurements of wound size reduction, percentage granulation tissue coverage, and extent of necrotic tissue clearance could not be obtained, and clinical response was therefore characterized qualitatively.

## 5. Conclusions

CODs hypothetically enhance autolytic debridement through engagement of endogenous enzymatic and cellular pathways, rather than passive conditioning of the wound environment. Based on the present observations, we propose that debridement may be functionally coupled with angiogenesis and granulation tissue formation, creating a self-reinforcing biological loop in which improved perfusion further supports tissue clearance. This integrated mechanism may help explain the observed ability of CODs to overcome biological stagnation in severely compromised wounds, including those with extensive necrosis, ischemia, or poor systemic reserve. Clinically, COD may reduce the need for sharp or surgical debridement or partially substitute it, offering a non-surgical therapeutic option in complex wound management.

These findings should be interpreted as hypothesis-generating and warrant further investigation in controlled prospective studies.

## Figures and Tables

**Table 1:** Patient Characteristics, Wound Etiology, and Prior Treatments Before Initiation of Copper Oxide Dressings.

Case	Age/Sex	Wound Type & Location	Comorbidities	Wound Duration Before COD	Prior Treatments
1	48 yr/M	Chronic venous leg ulcer	APLA; venous insufficiency; IVDU; fatty liver	8 yr	Multiple prior treatments; amputation proposed
2	60 yr/F	Distal femoral fracture infection → TFA stump	NIDDM	7 mo post-fixation; 1 wk post-revision	Failed TFA healing and revision; hip disarticulation considered
3	71 yr/M	Critically ischemic TFA stump (post-CABG)	Heart failure; pneumonia; NIDDM	1 wk post-amputation	Revision TTA → TFA; sutures removed before COD
4	19 yr/M	Electrical burn, left foot; toe gangrene	Previously healthy; 4-limb burns; hand amputation	10 d from injury	Betadine/chlorine wound care; toe amputations (~2 wk)
5 (2 limbs)	73 yr/M	Open femur fracture (L) + open knee dislocation (R)	NIDDM (25 yr)	2 wk post-injury	Washout; sharp debridement; external fixation; antiseptic care, antibiotic treatment.

**Table 2:** Wound Bed Features, Therapeutic Response, and Clinical Outcomes After Copper Oxide Dressing Application.

Case	Wound Type & Location	Baseline Wound Status	Perfusion	Infection/Cultures	Response	Outcome
1	Chronic venous leg ulcer	185 × 120 mm ulcer; mostly non-viable tissue; minimal granulation	Normal arterial perfusion	No clinical infection	Autolytic debridement; ~100% granulation at 4 wk	Complete closure at 18 mo
2	Distal femoral fracture infection → TFA stump	Central granulation with ischemic/necrotic margins	Palpable femoral pulse	MRSA cultured pre-COD; no active infection at start	Necrosis demarcation; ~95% granulation at 6 wk	Closed surgically (d52); sinus ~6 mo later due to osteomyelitis
3	Critically ischemic TFA stump (post-CABG)	>95% non-viable stump tissue; no infection signs	Weak femoral pulse	Pseudomonas in adjacent pressure sore cultures	Gradual necrosis clearance; dense granulation by d17	Death after 18 d (cardiopulmonary complications)
4	Electrical burn, left foot; toe gangrene	Extensive toe necrosis with dorsal involvement	No PVD; posterior tibial artery patent	Prior Enterococcus & Pseudomonas cultures; no infection at COD start	Autolytic debridement → robust granulation	TMA + STSG; complete graft take; good function
5 (2 limbs)	Open femur fracture (L) + open knee dislocation (R)	Extensive devitalized tissue without viable progression	CTA: major arteries patent bilaterally	Initial purulent exudate bilaterally(interpreted inflammatory)	Resolution of discharge; progressive granulation	STSG at 8–10 wk; 2-yr follow-up: intact skin; ambulates with walker

Abbreviations: COD—copper oxide dressing. MRSA—methicillin-resistant Staphylococcus aureus. CTA—computed tomography angiography. CABG—coronary artery bypass grafting. TFA—transfemoral amputation. TMA—trans-metatarsal amputation. STSG—split-thickness skin graft. PVD—peripheral vascular disease. wk—weeks. d—days. mo—months. yr—years.

## Data Availability

The original contributions presented in this study are included in the article. Further inquiries can be directed to the corresponding author.

## Abbreviations

The following abbreviations are used in this manuscript:

Abbreviation	Full term
APLA	Antiphospholipid Antibody
ATP7A	ATPase Copper-Transporting Alpha
ATP7B	ATPase Copper-Transporting Beta
ATOX1	Antioxidant Protein 1 (Copper Chaperone)
CD36	Cluster of Differentiation 36
CDP	Copper-Dependent Pathway
COD	Copper Oxide Dressing
CTR1 (SLC31A1)	Copper Transporter 1
ECM	Extracellular Matrix
FcγR	Fc Gamma Receptor
HIF-1α	Hypoxia-Inducible Factor 1 Alpha
ICU	Intensive Care Unit
IKK	IκB Kinase
IL-1β	Interleukin 1 Beta
IVDU	Intravenous drug user
MARCO	Macrophage Receptor with Collagenous Structure
MMP	Matrix Metalloproteinase
MMP-2	Matrix Metalloproteinase-2 (Gelatinase A)
MMP-9	Matrix Metalloproteinase-9 (Gelatinase B)
NF-κB	Nuclear Factor Kappa B
NPWT	Negative-Pressure Wound Therapy
PDGF	Platelet-Derived Growth Factor
TIMP	Tissue Inhibitor of Metalloproteinases
TIMP-1	Tissue Inhibitor of Metalloproteinases-1
TIMP-2	Tissue Inhibitor of Metalloproteinases-2
TNF-α	Tumor Necrosis Factor Alpha
VEGF	Vascular Endothelial Growth Factor
